# Seasonal stem growth analysis shows early stem growth of *Miscanthus* from high latitudes yields more biomass but stem traits negatively interact to limit seasonal growth

**DOI:** 10.3389/fpls.2025.1569235

**Published:** 2025-04-25

**Authors:** Paul R. H. Robson, Sarah Hawkins, Christopher L. Davey, John C. Clifton-Brown, Gancho Slavov

**Affiliations:** ^1^ Institute of Biological, Environmental and Rural Sciences (IBERS), Aberystwyth University, Aberystwyth, Wales, United Kingdom; ^2^ Department of Agronomy and Plant Breeding I, Research Centre for Biosystems, Land Use and Nutrition (iFZ), Justus Liebig University, Giessen, Germany

**Keywords:** bioenergy, biomass, energy crops, growth curves, *Miscanthus*, stem traits

## Abstract

High yielding perennial grasses are utilised as biomass for the bioeconomy and to displace fossil fuels. *Miscanthus* is a perennial grass used as a source of biomass but most of the cultivated crop is limited to a naturally occurring hybrid *M. × giganteus*. *Miscanthus* species originate from an extensive latitudinal and longitudinal range across Asia and thus have considerable potential to diversify the crop and improve yield. In previous studies stem morphological traits correlated strongly with yield in *Miscanthus* but little is known about how the development of stem growth may be optimised across the growth season. The aims of this study are to identify strategies to optimise seasonal growth duration and improve yield. To do this yield and seasonal stem elongation were measured from large numbers of diverse genotypes and functional data analysis used to characterise and compare the diverse perennial stem growth strategies. A diversity trial of over 900 genotypes was established in three replicates in the field at Aberystwyth, UK. Stem elongation was measured across the entire season for 3 consecutive years and the Richards growth function was fitted to model growth. Differentials, double differentials and integrals of the parameterised functions produced six growth characteristics, describing the growth rate, the timing and duration of the logarithmic growth phase and the integral of stem growth. Plants were also assessed for yield and moisture content. Growth traits from all plants in the diversity trial were moderately correlated, were correlated with biomass moisture content but less so to accumulated dry weight of biomass. Plants that grew for longer tended to have lower growth rates, but individual exceptions were identified. Plants with a similar duration of logarithmic growth achieved greater growth rates and harvestable yield if growth began earlier in the season and early season growth was mostly explained by latitude and altitude from which the accessions were collected. Stem growth traits were highly heritable and there was a significant effect of species on all growth characteristics. We discuss the possible interactions between growth and developmental control in perennials that may be exploited to improve yield in these crops.

## 1 Introduction

Increasing global temperatures are associated with increasing emissions from fossil fuels and levels of carbon dioxide in the atmosphere and therefore global efforts are being made to reduce emmisions. One approach has been to displace fossil fuel use using plant biomass which has the advantage of being produced by the fixation of atmospheric carbon dioxide over the short term. A range of biomass sources have been mooted including annual food crops, waste products and dedicated woody perennial biomass crops. The example of perennial biomass crops is particularly attractive because they usually have a good combination of characteristics that make them a good sustainable source of fixed carbon (Hodgson et al., 2024; Robson et al., 2020; Smeets et al., 2009). These sustainability characteristics include high annual yield, high energy output to low energy input ratios, the ability to be deployed at sufficient scale to impact on fossil fuel usage and the ability to be grown economically on land that may not be suitable for conventional food production.

Dedicated biomass crops include the perennial grass *Miscanthus* which is a C4 grass that unusually for C4 crops can grow successfully in temperate regions. Inter annual and inter-site variability in yield has been reported and yields range from 10 to 30 Mg DM ha^-1^ (Lewandowski et al., 2000) and were inversely correlated with latitude (Lesur et al., 2013). The crop is undomesticated and currently the commercial type is a natural triploid hybrid *M.* × *giganteus*. A number of traits have been investigated to improve yield and yield quality in *Miscanthus* including morphological traits such as canopy height (Jeżowski, 2008; Jones et al., 2015), phenological or developmental traits such as emergence, flowering and senescence (Fonteyne et al., 2018; Jensen et al., 2011; Robson et al., 2012), and traits effecting composition (Da Costa et al., 2014; Jorgensen, 1997). One approach discussed in (Long et al., 2006) is to increase photosynthesis of crop plants in general which may be achieved from different mechanisms including higher levels of light interception and increased efficiency of light conversion to biomass. A 60% increase in biomass yield was achieved in side-by-side trials of *Miscanthus* compared with maize due to *Miscanthus* producing a closed canopy approximately 4 weeks earlier and sustaining the canopy 4 weeks later than maize (Dohleman and Long, 2009). Recently other aspects of photosynthesis including improved light use efficiency have been highlighted as potential routes to increased yield (Taylor and Long, 2017).

The impact of canopy duration demonstrated by Dohleman and Long (2009) and the fact that *Miscanthus* is harvested annually, suggest increasing the growth season for higher annual light interception is a suitable trait for improvement. Improvements in yield would increase land use efficiency and CO_2_ fixation from the atmosphere and because high energy processes such as harvest occur once per year the energy balance will be even better. Light interception may be increased by canopy architecture, canopy duration or combination thereof. Our focus was on the duration of growth which we measured using stem elongation which captures early growth before canopy closure begins. The interaction between in particular early growth phenotypes and yield in *Miscanthus* has been reported previously (Fonteyne et al., 2018; Robson et al., 2019; Zub et al., 2012). The interaction between duration, yield and yield quality is complex. Early growth if killed by frosts or stressed by cold temperatures may result in non-productive heterotrophic growth depleting storage tissues. A similar scenario may be true for late season growth in which tissues that are not senesced may reduce the quality and sustainability of the crop. We hypothesise that a combination of long growth season and rapid growth will be traits of importance but it is not clear the extent to which such traits are aliased. A study of 5 high yielding plot-grown genotypes by Robson et al. (2019), showed that rapid growth rate and duration of the logarithmic growth phase were inversley related. We also do not know if early growth and early senescence are linked in *Miscanthus*. We hypothesise that the ability to grow at low temperatures early in the year in temperate environments, may be associated with low metabolic rates at peak season. Early growth may also reach a limiting factor such as intracanopy competition or a phenological stage such as flowering earlier thus curtailing the growth period. The extent to which different combinations of seasonal growth profiles are possible in *Miscanthus* species and the consequences for yield and yield quality was the subject of this study. We used a diverse collection of over 900 *Miscanthus* genotypes. We used stem elongation as a precise measure of seasonal growth across 3 years. We used Richard’s growth function (Richards, 1959) to model growth and generated characteristics of seasonal growth from differentiation and integration of parameterised growth functions. We used the growth characteristics to ask a number of questions 1) do growth traits vary between *Miscanthus* species and can variation in growth traits be explained by site of origin; 2) what are the interactions between different stem growth characteristics and between growth and yield or yield quality (moisture content); 3) are stem growth traits heritable and if so which species are most likely to contain useful extremes of stem growth traits; 4) do site of origin data and stem growth characteristics explain the ability to initiate early seasonal growth; and 5) using the large numbers of plants studied to create aggregations of similar durations can variation in the seasonal timing of growth demonstrate if early or late seasonal growth is preferential for yield. Our data demonstrate the species and geographic origins of useful variation in stem growth characteristics, we identify strategies for using seasonal stem growth to maximise yield and yield quality and discuss the potential for interactions between stem growth characteristics to limit yield.

## 2 Materials and methods

### 2.1 Trial site and treatment

Diversity trial: The trial site was established on a flat field (52°43′N, 4°03′W) near Aberystwyth on the west coast of Wales. The soil is classified as a dystric cambisol and a dystric gleysol depending on spatial variation in drainage (FAO, 1988) with a stone fraction (particles >2 mm) of approximately 15% (0–30 cm soil layer). Soil texture was 18% clay, 24% silt and 58% sand. The trial was in 3 fully randomised blocks except some genotypes were missing and had been replaced in some positions within the block to attempt to maintain a complete block structure. Plants were established from rhizome pieces on a 1.5 m grid during spring 2012.

Plant phenotyping in combination with markers has been commonly used in speciation of the *Miscanthus* genus (Chae et al., 2014). In the present study plants were assigned to species groups using a combination of expertise at collection sites and standard morphological characteristics as described below plus the identification of genetic groupings (Iacono et al., 2023). The following phenotypic traits were used to distinguish *Miscanthus* accessions collected directly in Asia or from secondary collections.

*M. sinensis* has compact non creeping rhizomes and forms tight tufts of shoots producing stems between 6-8 mm in diameter. *M. sinensis* flowers between July and October reaching a maximum height to the base of the panicle of 1 to 3 m depending on the growing conditions. The flowers appear greyish white with spikelets that end in a long and often twisted awn. Ripe seed is 0.5-0.8 mg and has a pale brown coat that is often rough (slightly crenulated).

*M. sacchariflorus* has creeping elongated rhizomes of which the meristems grow into shoots with a diameter of 5-35 mm and range from 1.5 to 5 m. The shoots are spaced 5-25 cm apart and therefore do not form tufts like *M. sinensis*, but rather spread to create monoclonal patches up to 2 x 2 m if space allows. The flowers appear silvery white, and the spikelets in the florets are awnless. The seed weighs 0.3-0.5 mg and is smooth and shiny.

*M. floridulus* is taxonomically similar to *M. sinensis* based on the rhizome and stem morphology, except it stays green in autumn and does not flower in northern Europe (UK). The flower axis is much longer it has longer racemes than *M. sinensis* leading to a much longer and bigger flower, but the spikelet awns are short and the seeds are smaller.

*M. lutarioriparius* is frequently indistinguishable from *M. sacchariflorus* using flower and rhizome morphology. This classification of *M. lutarioriparius* was based primarily on the origin in Central South China, Yangtze River basin.

There were four species groups identified within the collection that were the focus of the study: *M. sinensis*, *M. sacchariflorus*, *M. lutarioriparius*, and *M. floridulus*. There were also a small number of *M. sinensis* × *M. sacchariflorus* hybrids within the trial that were included as Hybrid1 (*M. sinensis* (♀) × *M. sacchariflorus* (♂)) and Hybrid2 (*M. sacchariflorus* (♀) × *M. sinensis* (♂)). The largest groups were *M. sinensis* (483 genotypes) and *M. sacchariflorus* (245 genotypes) (**Table 1**). The agreement between discrete groups identified from the assignment of species using standard morphological characteristics and the clustering of genetic markers into groups using discriminant analysis of principal components was 83% across the 4 species groups; *M. sinensis*, *M. sacchariflorus*, *M. lutarioriparius* and *M. floridullus*. The *M. sinensis* group had a substructure defined by 4 genetic groups labelled groups A to D. Site of origin data were available for many genotypes in the trial except for the majority of *M. sinensis* group B and D; therefore when examining associations to site of origin data these two groups were omitted.

**Table 1:** Seasonal growth characteristics of stem elongation from a diverse population of *Miscanthus* species and hybrids grown over 3 years.

		MaxGR	DayMaxGR	StartLogG	EndLogG	Duration	AUC
All plants (2014)	µ	1.2	189.7	140.6	234	93.4	14390.9
	σ^2^	0.5	24.1	23.5	27.4	34.3	4972.9
All plants (2015)	µ	1.4	199.5	162.5	236.4	73.9	27889.2
	σ^2^	0.6	19.1	21.4	20.6	17.7	11197.1
All plants (2016)	µ	2.1	181.4	143.7	217.9	74.2	32421.2
	σ^2^	0.8	15.3	16.6	19.9	20.2	9653.3
*M. floridulus*	µ	1.0	211.1	163.0	259.2	96.2	18441.9
(n=47)	σ^2^	0.4	19.2	27.7	22.8	33.3	6811.8
*M. lutarioriparius*	µ	2.8	175.1	136.7	213.4	76.7	44356.4
(n=79)	σ^2^	0.8	10.6	10.8	13.0	11.3	12857.7
*M. sacchariflorus*	µ	2.5	174.6	142.7	206.5	63.8	34292.3
(n=245)	σ^2^	0.7	10.0	9.7	12.5	10.1	8800.9
*M. sinensis* (A)	µ	1.5	184.6	140.9	228.2	87.4	26452.0
(n=127)	σ^2^	0.5	15.2	20.0	16.2	20.1	6676.0
*M. sinensis* (B)	µ	2.3	180.6	150.8	210.3	59.3	32805.7
(n=55)	σ^2^	0.4	9.9	12.1	9.9	10.0	4770
*M. sinensis* (C)	µ	1.9	182.0	143.0	221.0	78.0	31654.6
(n=245)	σ^2^	0.6	14.4	17.0	19.3	22.3	7466.1
*M. sinensis* (D)	µ	2.0	184.2	145.8	222.6	76.8	33308.0
(n=56)	σ^2^	0.4	17.3	20.1	17.1	14.4	5709.0
Hybrids1	µ	2.1	178.0	142.3	213.8	71.4	32093.6
(n=3)	σ^2^	0.4	7.5	8.7	10.8	13.1	3750.4
Hybrids2	µ	2.2	179.2	146.0	212.4	66.4	31213.7
(n=6)	σ^2^	0.5	14.2	16.4	13.9	5.4	6283.0

Variables are: maximum growth rate (MaxGR); day at which maximum growth rate was recorded (DayMaxGR), start of logarithmic growth (StartLogG), end of logarithmic growth (EndLogG), duration of logarithmic growth (Duration). Means and standard deviations are presented for all plants from 3 years and species groups and Hybrid1 (*M. sinensis* x *M. sacchariflorus*); Hybrid2 (*M. sacchariflorus* x *M. sinensis*) from 2016.

### 2.2 Growth measurements

Stem elongation was measured throughout the growth season usually approximately from May to October. Stem elongation was measured as the length of stem from the ground along the length of the longest stem to the point on the stem that was subtended by the ligule of the youngest differentiated leaf. Due to the large numbers of stems produced by some plants it was not always immediately obvious which was the longest stem and therefore a sample of the three longest stems was measured and the highest value of stem length recorded.

Plant biomass was measured as the weight of all above ground biomass (stems and leaves) from a cutting height of approximately 10 cm from the soil surface. A subsample of approximately 200 g of biomass was weighed shortly after harvesting and after being dried to constant weight at 60°C. The water content of the dried biomass of the subsample was calculated and used with the whole plant wet weight to estimate whole plant dry weight.

### 2.3 Meteorological factors

Daily climate data for the trial site were obtained from the Gogerddan weather station (52°25′N, 04°01′W), 500 m from the trial site. Data were recorded using a datalogger (Type CR10, Campbell, Leicestershire, UK). Heat accumulation, in degree days (°Cd), was calculated on a daily time step above a threshold temperature of 10°C, using equations described by (McVicker, 1946) using daily maximum and minimum air temperatures.

### 2.4 Data analysis and modelling

All analysis was completed using R (R Core Team, 2018) with some bespoke scripts. Functions were fit to individual plant data using the “nlsLM” function from the package “minpack.lm” (Elzhov et al., 2023). Stem elongation data were modelled using custom scripts to fit the four parameter Richards growth function (Equation 1), *x* is the explanatory variable such as Julian day, and *a*, *b*, *c*, and *d* parameters of fit.


(1) $$\frac{a}{1+b*exp^{{\left(-c*x\right)}^{\left(\frac{1}{d}\right)}}}$$

The parameters derived from individual plant data and the Richards function were used for further study. Parameterized functions were differentiated to identify the maximum growth rate (MaxGR) and when MaxGR occurred was estimated to the nearest Julian day (DayMaxGR). Thermal time was accumulated daily as degree days (°Cd) therefore when thermal time was used as an explanatory variable to calculate growth parameters the “Day” prefix was kept for consistency. The second differential was used to estimate the start and end of the logarithmic growth phase (StartLogG) and (EndLogG) respectively and the difference between the two was calculated to be the duration of the logarithmic growth phase (Duration). The final growth characteristic calculated was the integral of the curve (AUC).

Data were analysed for normality using the Shapiro Wilkes test from the R package “nortest”. Correlations used the “rcorr” function from the HMisc package (Harrell, 2016) and the “pearson” method to calculate the parameteric Pearson’s product-moment coefficient of correlation (r) and significance. The significant differences between the means of groups were compared by ANOVA. The numbers of each species were different and therefore a type III ANOVA was used to compare yield and growth characteristics between species. This was performed using the aov function and the Tukey’s HSD test from the R library ‘Agricolae’ (Mendiburu, 2017).

We conducted mixed linear model analyses using the lme4 package in R (R Core Team, 2018) including genotype and block as a random effect and used the resulting variance components to calculate broad-sense heritability (*H* ^2^) as:


$$H^{2}=V_{g}/(V_{g}+V_{e})$$

where V_g_ and V_e_ are the genetic and error variances, respectively (Falconer and Mackay, 1996).

## 3 Results

### 3.1 Variation in stem growth characteristics across Miscanthus species

Stem elongation was measured across 3 growth seasons (2014-2016) ensuring early emergence and asymptotic growth were captured to maximise the likelihood of a successful fit using Richards sigmoid growth equation. The equation was fit to individual plants to produce a parameterised equation for each. The differential, second differential and integral were calculated from the parameterised equations to generate 6 biologically meaningful growth characteristics for each plant which in the abbreviated form were MaxGR, DayMaxGR, StartLogG, EndLogG, Duration and AUC as described in Methods. Averaged values of MaxGR and AUC from all plants increased in each subsequent year but between year variations of other characteristics were less predictable. Julian day measurements (i.e. DayMaxGR, StartLogG and EndLogG) were latest in 2015 and Duration peaked and was similar in 2015 and 2016 (**Table 1**).

**Figure 1:**
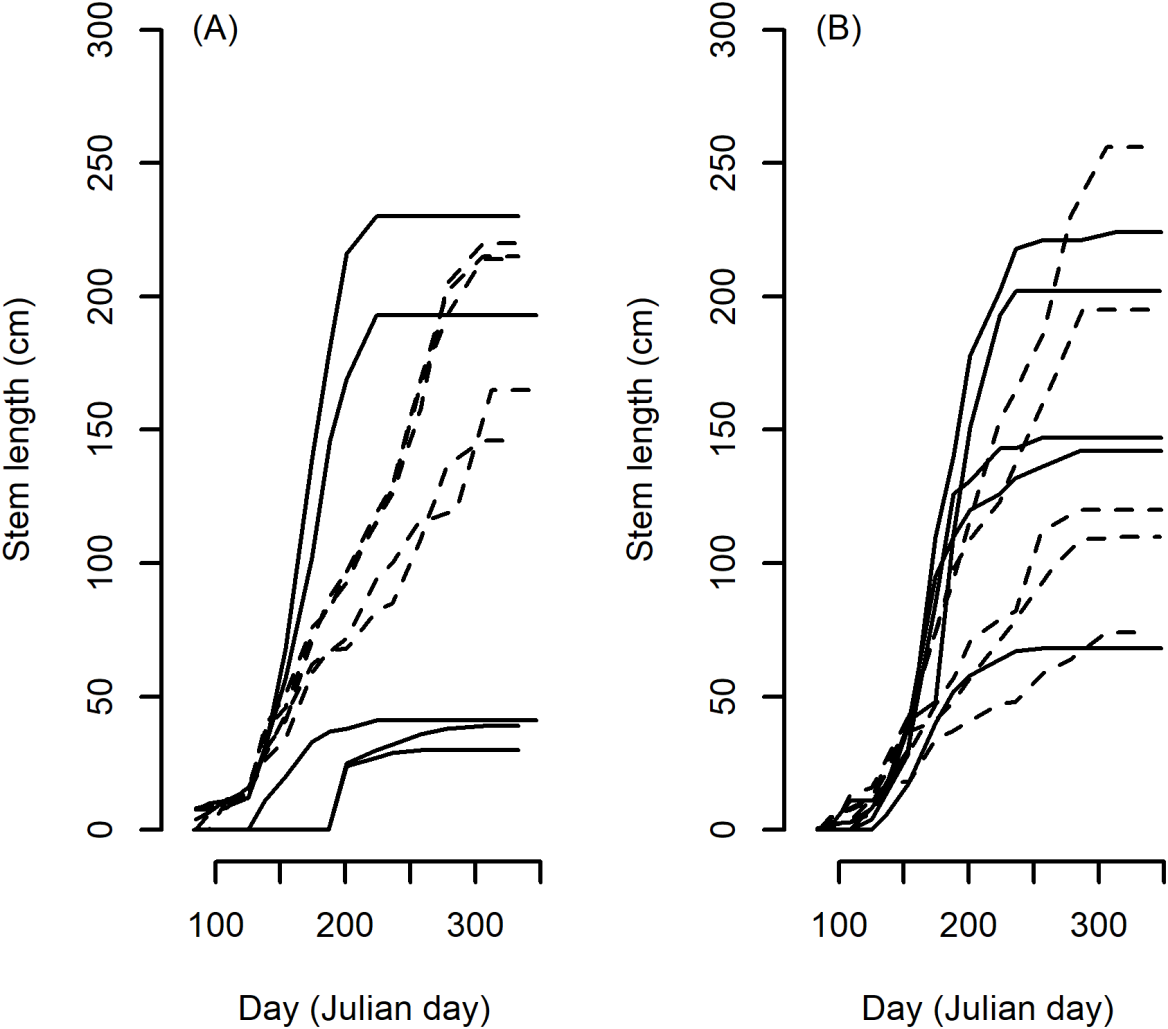
Stem elongation growth plots from 5 longest (dashed line) and 5 shortest (solid line) Duration plants of the two largest species groups in the trial, *M. sinensis* **(A)** and *M. sacchariflorus* **(B)** growing in 2016 demonstrating the large span of stem heights within plants of similar duration.

There was a significant effect of species on all growth characteristics and all biomass measurements (p < 0.001). The general trends between species are described below but there was considerable variation of interest within species. For example taking the very highest and lowest duration plants in the *M. sinensis* and *M. sacchariflorus* species groups produced examples of tall and short stemmed plants within both the short and long duration extremes (**Figures 1A, B**) particularly within the extremes of *M. sacchariflorus* species (**Figure 1B**). Some general trends were seen comparing averaged values and groups assigned by *post hoc* Tukey HSD test (not shown but **Table 1** includes means and standard deviations from 2016). Tukey HSD test assigned *M. floridulus* plants to a single species group with the longest duration and formed a similarly unique group with the lowest MaxGR. Above ground biomass accumulation was assessed from single plants in the two final years (2015 and 2016). In both years the species groups with the largest average per plant dry weights were *M. floridulus* and *M. sinensis* but in both years the hybrids between *M. sinensis × M. sacchariflorus* produced the highest averaged yields.

*M. floridulus* formed a group that was the latest to both enter and exit logarithmic growth. The late exit meant on average *M. floridulus* accumulated high levels of wet weight biomass but the biomass was of high moisture content. Despite having the lowest MaxGR the long Duration meant dry weight yields were similar to some other species groups that had faster growth rates such as the *M. sinensis* species group and were higher than *M. lutarioriparius* and *M. sacchariflorus*. *M. lutarioriparius* were assigned to a single species group with the fastest MaxGR and the highest integral of accumulated growth (AUC) because of early growth. The averaged day at which plants entered and exited the logarithmic growth phase was similar across the species groups in both years. In 2016 the *M. lutarioriparius* group started and ended the logarithmic phase of growth early in the season resulting in the lowest biomass accumulation in both years and low moisture contents (**Table 1**). In several of these growth characteristics *M. lutarioriparius* were not distinguishable from *M. sacchariflorus* species with the significant exceptions of growth rate and moisture content.

The *M. sinensis* group was assessed as both a single species group and as four genetically distinguishable groups labelled sinA to sinD. There were significant differences identified *post hoc* between the four genetic groups. The geographic origins of the genotypes from two groups, sinA and sinC, were largely known. The sinA genetic group came primarily from Southern Japan and South Korea, the sinC genetic group came primarily from Northern and mid-Japan. The maximum growth rate was significantly lower in the more Southerly genetic group. The sinA (Southerly) group entered log phase earlier than the sinC group but this difference was not statistically significant. However, the later exit from log phase by the sinA group was significant resulting in a significantly longer growth duration and significantly greater moisture content in the biomass harvested after winter.

### 3.2 Identifying species that contain extremes of seasonal growth traits

The numbers of the 4 different species within the trial were different but we assumed the sample was randomly picked from a larger species population. To examine which populations were the most likely source of extremes of growth traits the highest and lowest 5% for each trait were adjusted for group size of each species within the trial (**Table 2**). Plants with high MaxGR usually have low Duration and *vice versa* (**Table 1**). To examine this trend further two additional composite traits were included to identify the species most likely to exhibit a positive correlation between traits MaxGR and Duration. The composite traits were the ratio of MaxGR: Duration and the sum of the ranked values for the two traits (**Table 2**).

**Table 2:** Percentage of *Miscanthus* species present in a large diversity population that occur within the most extreme 5% of growth traits adjusted for numbers of species in sample.

	MaxGR	Day MaxGR	Start LogG	End LogG	Duration	AUC	MaxGR: Duration	Rank sum MaxGR+ Duration
High 5%								
*M. flor*	0	48.9	33.0	53.4	30.7	0	0	0
*M. lut.*	20.6	1.2	1.2	1.8	1.8	40.6	5.5	46.7
*M. sacc.*	9.9	0.3	1.5	0	0	4.6	10.7	2.1
*M. sin. (A)*	0	6.8	8.8	3.4	10.0	0	0.4	0
*M. sin. (B)*	0	0.8	2.3	0	0	0	3.1	0
*M. sin. (C)*	0.6	4.4	4.2	5.3	7.0	0.2	3.4	1.1
*M. sin. (D)*	0	4.9	5.7	6.5	1.6	0	0	0
Low 5%								
*M. flor.*	33.0	1.1	2.3	1.1	3.4	40.9	31.8	19.3
*M. lut.*	2.4	4.2	1.8	1.8	0.6	3.6	2.4	1.2
*M. sacc.*	0.9	5.3	0.3	10.2	4.1	1.9	0.2	4.6
*M. sin. (A)*	11.4	4.6	16.0	0.4	1.1	8.4	12.9	3.8
*M. sin. (B)*	0	1.5	0	1.5	16.2	0.8	0	6.9
*M. sin. (C)*	4.7	6.4	7.6	4.5	7.9	3.2	5.1	4.7
*M. sin. (D)*	0.8	5.7	4.1	2.4	0	0.8	0	3.3

Values represent plants of each of 4 species (*M. floridulus, M. lutarioriparius, M. sacchariflorus and M. sinensis;* the latter being split into 4 genetic groups) within the highest and lowest 5% of ranked trait values as a percentage of the individual species in the larger population.

*M. lutarioriparius* plants were most highly represented as a source of high growth rates, and high AUC plus the summed ranked score of MaxGR and Duration but were not well represented among the more extreme low trait values. *M. floridulus* was most likely to contain extremes, both highest and lowest, of growth traits. Being in both extremes was compatible with some of the phenotypes measured for example *M. floridulus* plants were most likely to be the latest, i.e. highest averaged Julian Day (and therefore be represented in the highest 5%) to start and end Log growth and the latest to achieve maximum growth rate, and as a consequence of much later growth low AUC. *M. floridulus* species were more highly represented in the traits low MaxGR and long Duration and were most likely represented in the lowest 5% of the two composite traits expressing the mostly undesirable combination of long duration and slow growth of this species in the trial. Plants of *M. sinensis* and *M. sacchariflorus* species were often well represented but not the most prevalent source of extreme trait values. Although notably *M. sacchariflorus* was most often represented in the group of plants that stopped log growth earliest (resulting in low moisture content of harvested biomass) and had the highest MaxGR: Duration ratio reflecting the high growth rate and short Duration of this species group (**Table 2**).

### 3.3 Seasonal growth trait heritability

Broad sense heritability (*H* ^2^) was calculated for growth characteristics from each of 3 years (**Table 3**). The *H* ^2^ values were mostly slightly higher in the later years. Two characteristics AUC and MaxGR consistently produced the highest *H* ^2^ (0.69, 0.77 and 0.81 for AUC data from 2014, 2015 and 2016 respectively; 0.71, 0.76 and 0.77 for MaxGR data from 2014, 2015 and 2016 respectively) and most other stem growth characteristics had a moderate to high heritability (0.38 to 0.71) across most years. Dry weight of aboveground biomass and its moisture content were assessed for two years (2015 and 2016). During these years *H* ^2^ values were moderate to high for moisture content (0.66 and 0.8 for data from 2015 and 2016 respectively) and moderate for dry matter (0.61 and 0.59 for data from 2015 and 2016 respectively).

**Table 3:** Broad sense heritability of seasonal stem growth and biomass traits from a field trial of diverse *Miscanthus*.

Trait	*H* ^2^ (2014)	*H* ^2^ (2015)	*H* ^2^ (2016)
MaxGR	0.71	0.76	0.77
DayMaxGR	0.38	0.59	0.66
StartLogG	0.54	0.54	0.57
EndLogG	0.51	0.64	0.71
Duration	0.6	0.57	0.65
AUC	0.69	0.77	0.81
MC	n/a	0.66	0.8
DM	n/a	0.61	0.59

Variables are: maximum growth rate (MaxGR); day at which maximum growth rate was recorded (DayMaxGR), start of logarithmic growth (StartLogG), end of logarithmic growth (EndLogG), duration of logarithmic growth (Duration), moisture content of harvested biomass (MC), weight of harvested biomass (DM).

### 3.4 Growth trait and yield correlations

Growth trait and dry weight yield (DW) correlations were tested using the combined data from all plants (**Table 4**). Most correlations were highly significant except StartLogG and either MaxGR or moisture content. Correlations with DM yield were low and correlations to the three traits expressed as Jd, DayMaxGR, StartLogG and EndLogG, were negative, all others were positive. Moisture content was negatively correlated with MaxGR and had similar positive correlations with EndLogG and Duration. All growth traits that were significantly correlated with MaxGR were negatively correlated.

**Table 4:** Growth and biomass yield correlations for a diverse population of field grown *Miscanthus* plants (2016).

Variables	1.	2.	3.	4.	5.	6.	7.
1. MaxGR	–						
2. DayMaxG	-0.40***	–					
3. StartLogG		0.81***	–				
4. EndLogG	-0.60***	0.87***	0.41***	–			
5. Duration	-0.58***	0.19***	-0.43***	0.65**	–		
6. AUC	0.87***	-0.44***	-0.26***	-0.46***	-0.23***	–	
7. MC	-0.67***	0.35***		0.55***	0.54***	-0.58***	–
8. DM	0.19***	-0.23***	-0.26***	-0.14***	0.08**	0.31***	0.11***

Variables are: maximum growth rate (MaxGR); day at which maximum growth rate was recorded (DayMaxGR), start of logarithmic growth (StartLogG), end of logarithmic growth (EndLogG), integral of growth curve (AUC), duration of logarithmic growth (Duration), moisture content of harvested biomass (MC), weight of harvested biomass (DM). Missing values were not significant: (> 0.1), significance level is denoted by symbol: (***, 0.001; **, 0.01).

Growth trait correlations were tested within species groups (data not shown). These were broadly similar to the correlations calculated using the entire dataset except coefficients were usually greater. Duration was always negatively correlated with MaxGR and was mostly very highly significant and highly correlated across all 3 years. Duration was always negatively correlated with StartExpG and ranged from very highly significant and highly correlated across all 3 years in the *M. sinensis* to very highly significant but moderately correlated in the *M. sacchariflorus* species group. Significant correlations between Duration and EndExpG were always positive. Correlations between DW and AUC were from the lowest R=0.45 (p<2x10^-16^) and R=0.48 (p<2x10^-16^) (*M. sacchariflorus* 2015 and 2016) to the highest 0.84 (p<2x10^-16^) and R=0.83 (p<2x10^-16^) (*M. floridulus* 2015 and 2016). MaxGR of all species groups was significantly correlated with DW; correlations were lowest in *M. sinensis* groups A to D (for example in 2016 values ranged from R=0.30, (p=7.6x10^-4^) to R=0.35 (p<2x10^-16^), in other species correlation coefficients were higher for example in the same year the highest value (R=0.68, p<2x10^-16^) was in *M. lutarioriparius*. Growth characteristics StartExpG EndExpG were both significantly and negatively correlated with DW. The highest correlations with moisture content were mostly with MaxGR, AUC and EndLogG. Moisture content was always negatively correlated with MaxGR and AUC but positively correlated with EndLogG. The highest coefficients of correlation with moisture contents were estimated from MaxGR of *M. sinensis* group C (R=-0.62, p<2x10^-16^ (2015); R=-0.73, p<2x10^-16^ (2016)) and AUC of *M. floridulus* (R=-0.64, p<2x10^-16^ (2015)).

### 3.5 Association between seasonal timing of growth and yield or maximum growth rate

**Figure 2:**
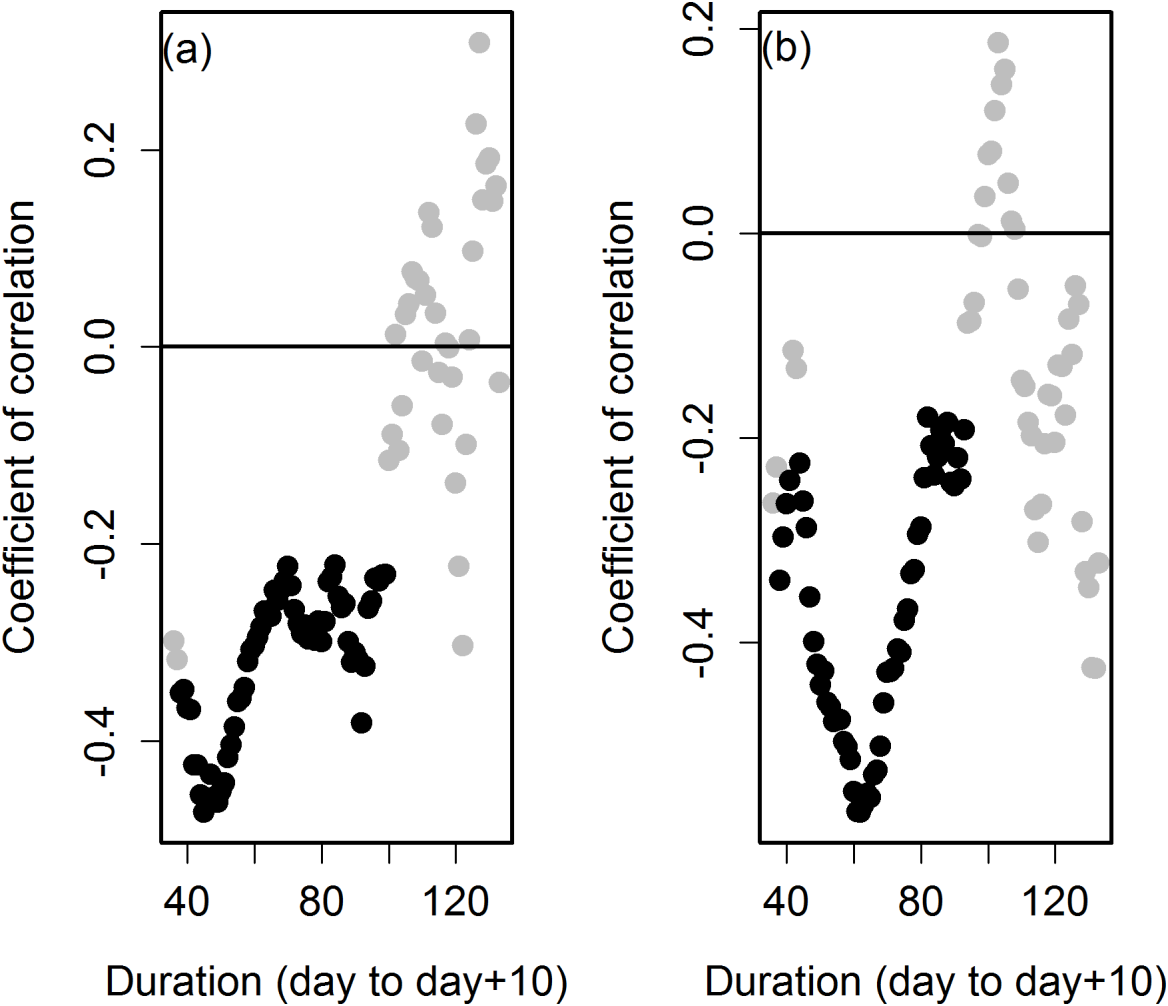
Correlation between day of year at which Duration began and either dry matter yield **(a)** or maximum growth rate of stem **(b)** for different durations of the logarithmic growth phase. Durations were binned (bin size = 10d) and significant and negative correlations occur between approximately 40-50 d to 100-110 days. Data are from all plants growing in 2016. Black symbols are significant correlations (P<0.05), grey symbols are non-significant correlations.

To test the impact on biomass accumulation of approximately similar lengths of Duration occurring at different times of year we tested the correlation between total dry weight biomass and StartLogG within bins of Duration. Bin size of Duration used was 3 d and up to 10 d across the entire range of short to long Durations. Any bins not containing 10 or more plants were discarded. The smaller bins examined down to as little as 3 d produced similar trends to the 10 d bins but with slightly more bins failing to meet the 10 plant threshold (data not shown). Correlations between DM and StartLogG were negative and moderate for 10 d Duration bins from approximately 40 - 50 d to approximately 100 - 110 d. Correlations from longer Duration bins became less or non-significant and fluctuated from negative to positive (**Figure 2a**). Two species groups *M. sinensis* and *M. sacchariflorus* had sufficient individuals to replicate the analysis and thereby reduce any potentially confounding species differences. The analysis using either species group was consistent with that performed using all data (data not shown).

A similar exercise examined the correlations between MaxGR and StartLogG across 10 d Duration bins (**Figure 2b**). With some exceptions the day at which the logarithmic phase began was not correlated with MaxGR; however, Duration is correlated with MaxGR. Therefore to control for the confounding effect of Duration, MaxGR from plants with similar length of Duration, but occurring at different times of year, was tested for correlation with StartLogG. Correlations between StartLogG and MaxGR were negative and moderate for Duration bins from approximately 40 - 50 d to approximately 90 - 100 d. Correlation coefficients between MaxGR and StartLogG were lower in value and non-significant from 10 day Duration bins longer than approximately 100 d.

The parameterised growth equations were used to calculate the Julian day and accumulated degree days at a 15 cm stem height (**Table 5**, shows Jd; trends from all 3 years, degree days (°Cd) estimations were similarly ranked and are not shown). There was a significant difference between species in the number of days and accumulated degree days to a stem elongation of 15 cm (p<2x10^-16^ for all 3 years). Trends were more consistent comparing the two final years of measurement. Genotypes of *M. sinensis* and *M. lutarioriparius* species were the earliest to reach 15 cm and *M. floridulus* the latest. Comparing the two species groups *M. sinensis* and *M. sacchariflorus*, *M. sinensis* was consistently just under two weeks earlier than *M. sacchariflorus*.

**Table 5:** Accumulated Julian days to 15cm stem length from a diverse population of *Miscanthus* species and modelled relationship to coordinates of collection site.

	2014				2015				2016			
	Mean Jd.	T_HSD_	Geo.	*r(P-value)*	Mean Jd.	T_HSD_	Geo.	*r(P-value)*	Mean Jd.	T_HSD_	Geo.	*r(P-value)*
All plants			**Lat**	**0.01 (2.9x10^-3^)**			**Lat**	**0.09 (<2.2 x10^-16^)**			**Lat**	**0.06 (<2.2 x10^-16^)**
*M. flor.*	126.2 +/-3.9	ab	**Lat**	**0.74 (1.9x10^-3^)**	158.7 +/-3.6	a	**Alt**	**0.25 (7.7x10^-8^)**	140.8 +/-2.6	a	Lat(*^2^Alt)	**0.26 (3.7x10^-7^)**
*M. lut.*	121.4 +/-2.0	b	Long	0.02 (0.13)	142.0 +/-1.4	b	**Long**	**0.03 (4.5x10^-2^)**	125.5 +/-0.7	c	Alt	0.004 (0.69)
*M. sacc.*	136.7 +/-0.7	a	Long	-2.7x10-3 (0.76)	144.7 +/-0.6	b	**Alt**	**0.02 (4.4x10^-3^)**	135.8 +/-0.3	b	**Alt**	**0.04 (9.7 x10^-6^)**
*M. sinensis* (group A)	119.5 +/-1.7	b	**Long**	**0.08 (7.8x10-4)**	134.6 +/-1.6	c	**Long**	**0.08 (7.0x10^-6^)**	126.1 +/-0.7	c	**Long**	**0.10 (7.2x10^-7^)**
M. sinensis (group C)	110.4 +/-1.0	b	Alt	-6.6x10^-3^ (0.93)	127.6 +/-1.1	c	**Alt**	**0.04 (9.7x10^-4^)**	120.2 +/-0.5	c	**Alt**	**0.03 (2.5x10^-3^)**

Average and standard errors are presented for selected species groups; Geo. in bold is the most significant coordinate, latitude (Lat), longitude (Long) or altitude (Alt), r; adjusted squared coefficient of regression; *^2^ Alt also had r = 0.26 (but lower than Lat to 3 sig fig.).

### 3.6 Site of origin associations with seasonal growth traits

When all plants were used in regression models latitude at collection site explained variation in Jd and accumulated degree day to 15 cm and, compared to other site location and meteorological data, the models were most significant when latitude was used as the explanatory variable (**Table 5**). The coefficients of regression were low to moderate and the slope indicated genotypes from lower latitudes reached 15 cm earlier in the year or at a lower value of accumulated degree days. Using data from growth years 2015 and 2016 *M. sinensis* growth to 15 cm was most significantly explained by altitude and the slope indicated genotypes from lower altitudes reached 15 cm earlier in the year or at a lower value of accumulated degree days. Regression models of growth from other species were less consistent but significant models included longitude and altitude as the most significant geographical parameter (**Table 5**).

## 4 Discussion

Growth traits were measured across a diverse collection of perennial *Miscanthus*. Improved understanding of phenology and the regulation of growth is vital in adapting crops to different regions and in predicting the impact of environmental change on ecosystems. In long season perennial biomass crops, such as *Miscanthus*, growing in temperate regions the induction of flowering and senescence is associated with the cessation of growth and limits further yield accumulation (Jensen et al., 2012; Robson et al., 2013b). However, little is known about the seasonal growth dynamics in this species. Zub et al. (2012) compared a small number of genotypes and demonstrated that yield in *Miscanthus* was associated with high growth rates. In a previous study of 5 high-yielding genotypes growing in plots Robson et al. (2019) demonstrated an inverse relationship between maximum growth rates and duration of logarithmic growth. Here we extend those observations to a large number of diverse species of *Miscanthus* from which there was a significant effect of species on growth traits.

The correlations between most growth traits and correlations between growth traits and biomass were highly significant but unlike previous studies (Robson et al., 2019) coefficients were low when relating growth traits with biomass. Low coefficients may reflect the high level of within population variance as was reported in flowering studies of *Miscanthus* (Yan et al., 2012). This was in agreement with the analysis at the individual species level which resulted in high values of coefficients as reported above. The correlation coefficients may indicate that the population represents many potential seasonal growth trajectories toward final accumulated biomass without the confounding influence of intraspecific competition and thus represents an ideal experiment to understand the genetic control of seasonal development. Growth traits were highly correlated with accumulated biomass in competitive plots (Robson et al., 2019); which contrasts with the use of spaced plants in the current trials and may indicate growth traits generate more significant correlations with yield when there is seasonal competition for resources.

In general stem traits correlate well with final harvested yield, this being the product of both stem height and stem number but these two traits are to some extent confounded. High yield may be derived from low stem numbers and long thick stems or large stem numbers of thin and shorter stems (Robson et al., 2013a). Accepting the yield correlation of these confounded traits our results allow us to better define the optimal growth trajectories for yield and yield quality, the latter in this instance was assessed as moisture content. From such significant diversity it may be possible to identify which species and where in the natural environment are *Miscanthus* plants likely to be found with growth trajectories of most interest.

Growth rate within ecosystems has been defined within one or a combination of 3 broad strategies: competitive, stressed or ruderal (C-S-R) (Grime, 1977). *Miscanthus* comprises highly diverse species and many species range over very different geography. One exception is *M. lutarioriparius*, the tallest growing *Miscanthus* species endemic to central China (Yan et al., 2012). *M. lutarioriparius* occupies a relatively narrow distributional range and ecological niche growing vigorously along seasonally flooded river banks and lake shores in central China (Chen and Renvoize, 2006). Flooded riverbanks represent a ruderal habitat in which disturbance is an important factor (Kyle and Leishman, 2009). The C-S-R classification (Grime, 1977) summarises ruderal strategies to include plants that are short-lived, rapid maximum growth rate, a short investment in leaves and small investment in seed. The growth rate component of this classification appears consistent with growth traits in *M. lutarioriparius* which started growth early, grew rapidly and for a short period only (**Table 2**). However, *M. lutarioriparius* species yielded the lowest biomass and consequentially, unless several harvests may be taken per growth year, segregation in linked growth parameters, such as Duration and MaxGR, will be required to maximise the yield potential. The tradeoff between growth rate and Duration parallels the general tradeoff observed between survival traits and growth rate as exemplified by the tradeoff in growth rate and cold tolerance observed in trees (Loehle, 1998). The problem of how to break the link between growth rate and Duration may benefit from studies of unusual exemplars. Identifying species and environments allows general trends to be identified for further targeted collections but as illustrated in **Figure 1** the *Miscanthus* population examined contains extremes of phenotypes and represents a suitable resource to examine genetic linkage to different combinations of growth traits within the population.

When growing in temperate regions the high yield achieved by the commercial type *M.* × *giganteus* is attributed in part to cold tolerant C4 photosynthesis (Naidu et al., 2003) and a long growth duration during which more light may be intercepted (Dohleman and Long, 2009). Early seasonal growth of *Miscanthus* will allow the canopy to potentially intercept more light throughout the season and may provide a competitive head start against certain weeds. Expression of reactive oxygen scavenging molecules and water soluble carbohydrates associated with chilling stress have been reported in early-growing, cold-tolerant *Miscanthus* (Fonteyne et al., 2018). In this study, when combining plants of similar Duration, significant correlations between biomass yield or MaxGR and the onset of logarithmic growth (StartLogG) were always negative (**Figure 2**). This suggests seasonally earlier growth was positively correlated with dry biomass yield within diverse *Miscanthus* with very similar Duration. This is consistent with a study of canopy duration which suggested *Miscanthus* that grew earlier developed more yield (Robson et al., 2013a) and is consistent with a study of the seasonal timing of bud flush and biomass yield in Willow (Ronnberg-Wastljung and Gullberg, 1999). When considering where such early growing plants may be obtained there was genotypic variation in the time at which stems reached 15 cm by up to an average of 20-30 days depending on the year of study (**Table 5**). The Jd at 15 cm stem elongation was significantly correlated with geospatial coordinates. When all plants were included, latitude was the most significant factor but when individual species were considered in isolation many significant models included longitude and altitude. Significant coefficients were all positive suggesting plants that originated from lower altitudes and southerly latitudes achieved more rapid early growth in our trial.

It may be assumed that a combination of high MaxGR and long Duration would produce more harvestable biomass. When the sum of ranked values of MaxGR and Duration traits was compared and adjusted for species sample size, *M. lutarioriparius* appeared to be a good source of both traits but this was primarily driven by very high growth rates (**Tables 1**, **2**) and the resultant plants were low yielding due to the relatively short Duration (**Table 1**). Single species may not contain the optimal combination of traits and thus interspecific hybrids, which bring together evolutionarily less common combinations of growth traits, may be required. The most widely commercially grown *Miscanthus* is a natural hybrid between *M. sinensis* and *M. sacchariflorus* that occurred in a sympatric region (Nishiwaki et al., 2011). From the perspective of growth traits *M. lutarioriparius* represents an extreme version of *M. sacchariflorus* thus producing wide hybrids of *M. lutarioriparius* and *M. sinensis* may produce suitable high yielding progeny. However this is but one of many traits that may be optimised and there is much interest in utilizing the high genetic variation present within the widespread native populations of *M. sinensis* (Clifton-Brown et al., 2008; Stewart et al., 2009) in order to develop intraspecific stress-resistant, high-yielding lines for hybrids. We demonstrate here that considerable variation exists in *M. sinensis* for growth traits including long season and high growth rate genotypes (**Figure 1**) thus suggesting the possibility of readily combining potentially favourable stress and growth traits within one species may be possible.

The two *M. sinensis* populations A and C which contained sufficient site of origin data for further analysis had broadly similar growth characteristics including timing or early season growth; however, group C which predominantly originated from North and mid-Japan consistently grew to 15 cm earlier than group A which predominantly originated from South Japan and South Korea. The timing of early growth was significantly correlated with altitude in the more Northerly groups and Longitude in the more southerly group, the latter perhaps reflecting the longitudinal spread from Japan to South Korea (**Supplementary Figure 1**). Previous studies have shown a multiplicity of climatic variable explained flowering in *Miscanthus* from similar geographical regions (Jensen et al., 2011) and flowering will likely impact a subset of stem growth rate characteristics described here. Therefore, it is likely that more complex growth models parameterized for averaged meteorological conditions at the sites of origin will be needed to better explain the origins of stem growth characteristics.

Whether by intra- or interspecific recombination, combining multiple traits is a challenge that will likely be mostly achieved through the application of genomic selection. Such models may be developed from large diverse trials exhibiting rapid decay in linkage disequilibrium (Gaut and Long, 2003). Previous mapping and association studies in *Miscanthus* have focused mostly on traits that describe harvested biomass or components of biomass such as stem numbers, plant height and diameter (Atienza et al., 2003; Dong et al., 2018; Gifford et al., 2015; Nie et al., 2016) a few have included phenological traits (Jensen et al., 2012; Slavov et al., 2014). Little is known about function-valued traits as defined by Pletcher and Geyer (1999) that describe traits as a function of some independent and continuous variable such as time or temperature. Such traits are likely to be particularly impactful for crops in which the harvested product is largely the integral of seasonal growth such as is the case for biomass yield in *Miscanthus*. Understanding the inheritance of traits is important in effective parental selection. Here we showed stem growth traits are moderately heritable (**Table 3**).

Perennial plants retain vegetative meristems after flowering and we propose that perennials prioritise replenishment of vegetative meristems for growth in subsequent years before flowering. In a study of UK-grown *Miscanthus*, rhizome declined in early growth and was replenished later in the season (Beale and Long, 1997). We propose the duration of growth is adjusted in accordance with the rate of growth through replenishment of rhizome. We anticipate that breeding for higher growth rate will be desirable. However, a faster growing plant may not necessarily yield more as was exemplified by the species group *M. lutarioriparius* (**Tables 1**, **2**). Within the same environment plants with two different growth rates may achieve the same yield if the slower plant is still able to harvest sufficient resources to replenish vegetative meristems and trigger flowering and senescence before seasonal climatic conditions limit further growth. This seasonal limit may explain why correlations between yield and growth rate are lower in spaced plants because resource capture may be completed in less competitive plants, such plants would otherwise not complete their growth cycle within competitive plots. Faster growth, if it results in earlier seasonal cessation in growth, will likely be highly beneficial from the perspective of crop quality such as a lower moisture content (**Table 4**) which improves the efficiency of transportation and some conversion processes (Lewandowski et al., 2003).

We have demonstrated the enormous variation in growth traits available for development of *Miscanthus* as a biomass crop. This variation is biologically meaningful in that it is consistent with some ecological models such as the high MaxGR and short growth durations in ruderal environments identified in *M. lutarioriparius*. In general latitude and altitude appear significant correlates with earlier growth and this phenotype was strongly associated with improved yield compared to later seasonal growth. We demonstrate that seasonal growth traits are highly heritable in mature plants and show a consistent negative interaction between MaxGR and Duration. This negative interaction may in part explain the reduced growth phenomena of sugarcane in which radiation use efficiency declines seasonally due to a decline in specific leaf nitrogen, sugar feedback to photosynthesis or increased respiration (VanHeerden et al., 2010). Because annual plants evolved from perennials it would be expected that annuals may have retained some of the control exerted by perennials and a better understanding of the seasonal control of growth in perennial species may help better understand developmental control in annual plants. For example Target Of Rapamycin (TOR) is a master regulator of growth and development. Manipulating expression of TOR in transgenic *Arabidopsis* produced a similar pattern of reduced growth rate and delayed transition to flowering and longer growth duration (Ren et al., 2012) as was seen in the *Miscanthus* trial reported here. We have identified that the negative correlation between growth rate and duration in *Miscanthus* may be partially uncoupled but with species exemplified by large differences in growth traits it is likely that interspecific wide hybrids will be the most suitable route to superior trait combinations. Identifying the mechanism(s) resulting in seasonal growth limitation and potentially uncoupling linked traits through genotypic variation in this phenomenon will produce large increases in yield in *Miscanthus* and other perennial and annual plants.

## Data Availability

The original contributions presented in the study are included in the article/**Supplementary Material**. Further inquiries can be directed to the corresponding author.

## Supplementary material

Supplementary Figure 1 Area of distribution of Miscanthus species *M. sinensis* genetic groups A and C.
