# Supplementary figures and images for: Seasonal stem growth analysis shows early stem growth of Miscanthus from high latitudes yields more biomass but stem traits negatively interact to limit seasonal growth
Source: Front Plant Sci. 2025 Apr 25;16:1569235. doi: 10.3389/fpls.2025.1569235 (PMC12061675; doi:10.3389/fpls.2025.1569235)

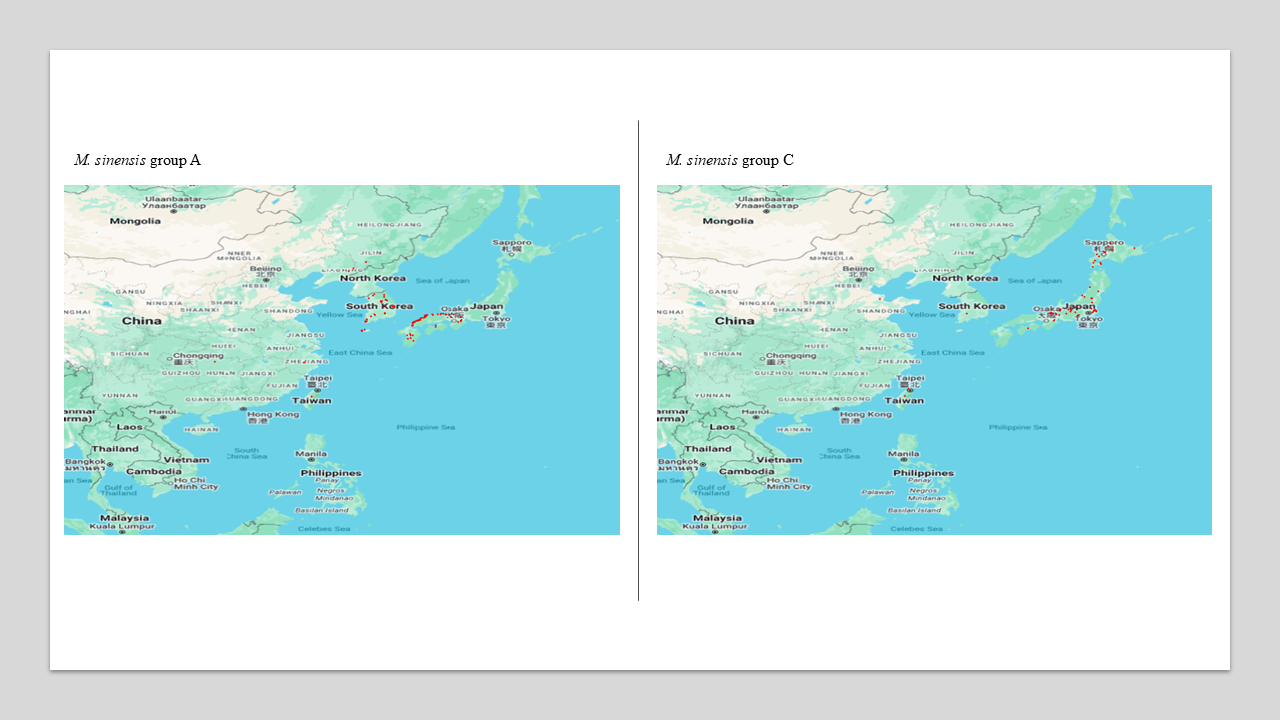

Supplement: Supplementary Figure 1 — Area of distribution of Miscanthus species M. sinensis genetic groups A and C. [file Image1.tif]
